# Gut Mechanisms Linking Intestinal Sweet Sensing to Glycemic Control

**DOI:** 10.3389/fendo.2018.00741

**Published:** 2018-12-04

**Authors:** Denise Kreuch, Damien J. Keating, Tongzhi Wu, Michael Horowitz, Christopher K. Rayner, Richard L. Young

**Affiliations:** ^1^Faculty of Health and Medical Sciences & Centre of Research Excellence in Translating Nutritional Science to Good Health, Adelaide Medical School, The University of Adelaide, Adelaide, SA, Australia; ^2^College of Medicine and Public Health, Flinders University, Bedford Park, SA, Australia; ^3^Nutrition and Metabolism, South Australian Health and Medical Research Institute, Adelaide, SA, Australia

**Keywords:** intestinal sweet taste receptors, L-cells, glucose transport, SGLT-1, glycemic control, type 2 diabetes mellitus

## Abstract

Sensing nutrients within the gastrointestinal tract engages the enteroendocrine cell system to signal within the mucosa, to intrinsic and extrinsic nerve pathways, and the circulation. This signaling provides powerful feedback from the intestine to slow the rate of gastric emptying, limit postprandial glycemic excursions, and induce satiation. This review focuses on the intestinal sensing of sweet stimuli (including low-calorie sweeteners), which engage similar G-protein-coupled receptors (GPCRs) to the sweet taste receptors (STRs) of the tongue. It explores the enteroendocrine cell signals deployed upon STR activation that act within and outside the gastrointestinal tract, with a focus on the role of this distinctive pathway in regulating glucose transport function via absorptive enterocytes, and the associated impact on postprandial glycemic responses in animals and humans. The emerging role of diet, including low-calorie sweeteners, in modulating the composition of the gut microbiome and how this may impact glycemic responses of the host, is also discussed, as is recent evidence of a causal role of diet-induced dysbiosis in influencing the gut-brain axis to alter gastric emptying and insulin release. Full knowledge of intestinal STR signaling in humans, and its capacity to engage host and/or microbiome mechanisms that modify glycemic control, holds the potential for improved prevention and management of type 2 diabetes.

## Introduction

It is now widely recognized that the gastrointestinal tract is a major determinant of metabolic homeostasis, and the largest endocrine organ of the body. This is due to the diversity and wide signaling repertoire of the gastrointestinal enteroendocrine cells (EECs) which can, collectively, release over 30 different peptide hormones and neurotransmitters (1). To subserve this signaling function gastrointestinal EECs are configured either as “open” cells—possessing long, slim, finger-like extensions on their apical side to sense the luminal milieu and, in turn, release signaling molecules, or as “closed” cells which do not access the lumen, but can respond indirectly to luminal content (2). EEC have classically been sub-divided according to their hormone or transmitter content, and regional location within the gastrointestinal tract. However, the substantial overlap in transcriptional expression and subcellular stores that has recently been identified now supports a more heterogeneous EEC population (3, 4).

## EECs Respond to Intestinal Carbohydrates

Exposure to luminal glucose generates signals that have a profound influence on intestinal motor and absorptive function. These signals include release of the gut peptides glucose-dependent insulinotropic polypeptide (GIP) from K-cells located in the proximal intestine and glucagon-like peptide-1 and 2 (GLP-1, GLP-2) from L-cells located in more distal regions of the intestine (5, 6), and release of the bioamine serotonin (5-HT) from enterochromaffin (EC) cells located throughout the gastrointestinal tract (7–9). GLP-1 and GIP, the “íncretin” peptide hormones, are degraded rapidly upon release by the ubiquitous enzyme dipeptidyl peptidase-IV and neutral endopeptidase, with <50% of secreted hormone entering the circulation. However, they powerfully augment glucose-dependent insulin release in response to an enteral glucose load (in comparison to an intravenous isoglycemic glucose load) (10, 11). GLP-1 and 5-HT also activate GLP-1 and 5-HT_3_ receptors on intestinal vagus nerve endings as key signals in the “gut-brain axis,” which, in turn, triggers vagal reflexes to slow the subsequent emptying of carbohydrate from the stomach, and induce satiation (12, 13). Accordingly, the release of GLP-1, GIP, and 5-HT is crucial to the regulation of postprandial glycemia. In contrast, GLP-2, which is co-released with GLP-1, is intestinotrophic and a potent signal to upregulate the expression and function of the primary intestinal glucose transporter, sodium-glucose cotransporter-1 (SGLT-1) (14).

## Sweet Taste Machinery

### Lingual Sweet Taste

All known sweet tastants, including hexose sugars, D-amino acids, sweet proteins (such as monellin and thaumatin), and low-calorie sweeteners (LCS) are sensed by a single broadly-tuned sweet taste receptor (STR), comprised of a heterodimer of class C, G-protein coupled receptors, T1R2, and T1R3 (15). In lingual sweet taste cells, where sweet taste transduction has been most fully characterized, the interaction of sweet tastants with STRs initiates dissociation of the G-protein, gustducin, into Gα and Gβγ subunits and activation of phospholipase C β_2_ (PLCβ_2_); intracellular Ca^2+^ is then released from inositol 1,4,5-trisphosphate-sensitive (IP3) stores, leading to opening of the melastatin type-5 transient receptor potential cation channel (TRPM5) to sodium influx [for review, see (16)]. Increases in intracellular Na^+^ and Ca^2+^ then depolarize the basolateral membrane and, via 5-HT and ATP-dependent pathways, activate intermediary taste cells and chorda tympani and glossopharyngeal nerves that convey taste information centrally to the insular cortex [for review, see (17)] (18–20).

### Intestinal Sweet Taste

STRs are well-described on subsets of EEC in the proximal small intestine, with evidence of STR-equipped K-cells, L-cells, and EC cells in humans (21, 22). STRs are also documented widely in metabolic tissues that sense and respond to carbohydrates, such as pancreatic β-cells, hepatocytes, adipocytes, and hypothalamic neurons [for review, see (23, 24)]. Expression of intestinal STR, like many GPCR, is of low magnitude, and optimally detected with high sensitivity SYBR-based PCR approaches rather than Taq-based PCR. Evidence in rodents, and in human cells and tissues, provides strong support that intestinal STRs function as upstream sensors linked to the release of GLP-1 from L-cells, and 5-HT from EC cells, and genetic deletion of T1R3, or pharmacological blockade of STRs with lactisole, decreases glucose and LCS-evoked GLP-1 and 5-HT release (21, 25–27). This is also true for GLP-2 release, which is STR-dependent in rodents (28, 29) and inhibited by the murine STR inhibitor, gurmarin (30).

Clinical studies have also reported acute effects of LCS to augment GLP-1 release in the presence of glucose and have shown a dose-dependent effect of lactisole to attenuate glucose-induced GLP-1 release in healthy subjects (31–34). Despite this, the balance of clinical evidence indicates that, at least in acute settings, LCS do not contribute substantially to the circulating pool of GLP-1 in humans (35–37).

### Interplay Between STRs and SGLT-1 Can Regulate Glycemic Responses

Enterocytes account for around 90% of all intestinal epithelial cells and are polarized cells consisting of apical and basolateral membrane domains (38). These cells transport nutrients from the gut lumen to the circulation, and for glucose, apical SGLT-1 is the primary intestinal glucose transporter in both humans and animals. SGLT-1 is expressed primarily in the small intestine with highest density in the jejunum followed by the duodenum and then ileum (39, 40). SGLT-1 enables glucose absorption by co-transporting sodium along the electrochemical gradient established by the basolateral sodium-potassium ATPase (38, 41). Glucose then enters the systemic circulation via the facilitative monosaccharide transporter, GLUT2, located on the basolateral membrane of enterocytes; GLUT2 is bidirectional and capable of moving glucose in or out of enterocytes depending on glucose concentration gradients (38).

Importantly, transport of the monosaccharide substrates of SGLT-1 (e.g., glucose and galactose) triggers incretin hormone secretion (20), an action attenuated when SGLT-1 is pharmacologically inhibited with the competitive antagonist phlorizin, or absent through genetic deletion in rodents (42, 43). Our group provided the first evidence that SGLT-1 substrates, even if not metabolized (such as the glucose analog 3-O-methyl-glucose, 3-OMG), have the capacity to stimulate GLP-1 and GIP secretion in humans (44). We have also established that SGLT-1-based transport is critical for *ex vivo* release of GLP-1 in human ileum, while blocking SGLT-1 with phlorizin or replacing extracellular Na^+^ with N-methyl-D-glucamine abolishes this response (26).

**Figure 1:**
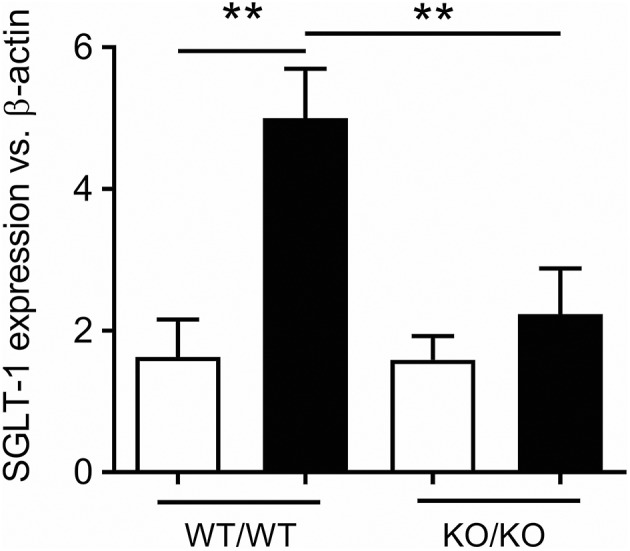
STR-dependence of SGLT-1 expression in mice. Increased jejunal expression of SGLT-1 mRNA in 10 week-old control (WT/WT) mice gavaged for 4 days with sucralose (black bars) compared to water (white bars), and to mice homozygous for both *Tas1r2* and *Tas1r3* genes (KO/KO). Breeding pairs of mice homozygous for the *Tas1r2* or *Tas1r3* gene (129X1/SvJ mice backcrossed for at least 3 generations with C57BL/6 mice) were provided by Prof Charles Zuker (University of California, San Diego, USA). Mice homogenous for each gene were then paired to produce mice heterozygous for *Tas1r2* and *Tas1r3*. These mice, in turn, were paired to generate mice heterozygous, homozygous, and wild-type for both genes. From these mice, double homozygous (KO/KO) and wild-type littermate controls (WT/WT) were the subject of gavage experiments. Ten-week old male mice (*N* = 5 per group) maintained under standard housing and diet conditions in the SA Pathology Animal Care Facility were gavaged twice daily with 100 mg sucralose (Redox Chemicals, Minto, NSW Australia) in 200 μL water, or 200 μL water, at 0800 and 1800 over 4 days. These mice were fasted overnight then humanely killed at 0800, total RNA extracted from the jejunal mucosa, and real-time RT-PCR performed using primer assays for SGLT-1 (QT00112679) and β-actin (QT01136772, Qiagen, Sydney, NSW Australia) relative to expression of β-actin, as described (49); SGLT-1 expression was compared between groups and gavage regime by analysis of variance (ANOVA), adjusted for multiple comparisons by Holm-Sidak's correction (GraphPad Prism 7.02, San Diego, CA, USA). This experiment was approved and performed in accordance with guidelines of the Animal Ethics Committees of The University of Adelaide and SA Pathology (Adelaide, Australia). Data is shown as Mean ± SEM; **P < 0.01. We thank Prof Charles Zuker for generously supplying the homozygous *Tas1r2* and *Tas1r3* mice.

**Figure 2:**
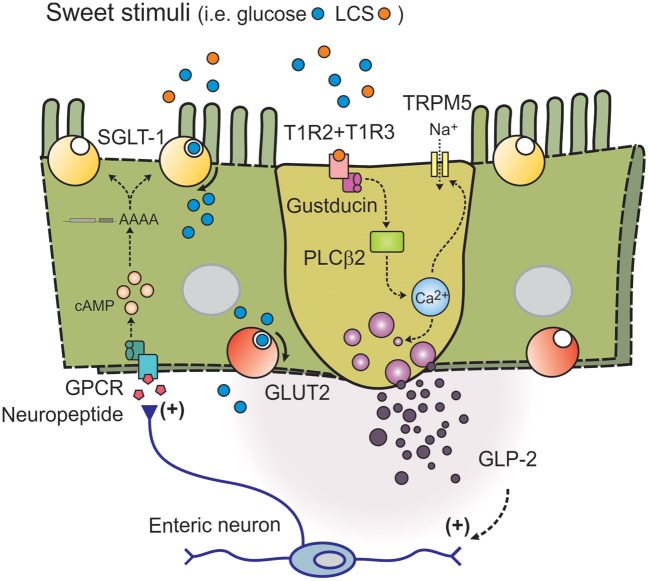
Model of intestinal sweet taste sensing and signaling effectors. Sweet stimuli, including LCS, bind to STR comprised of a heterodimer of G-protein coupled receptors T1R2 and T1R3. Upon receptor binding an intracellular signaling cascade is activated, initiated by dissociation of G-protein gustducin into Gα and Gβγ subunits and activation of phospholipase C β_2_ (PLCβ_2_); intracellular Ca^2+^ is then released from inositol 1,4,5-trisphosphate-sensitive stores, leading to opening of the melastatin type-5 transient receptor potential cation channel (TRPM5) to sodium influx. Increases in intracellular Na^+^ and Ca^2+^ then depolarize the basolateral membrane, to facilitate release of peptide hormones such as GLP-2. GLP-2 may then trigger an enteric neuron pathway to release an unknown neuropeptide at nearby absorptive enterocytes leading to adenylate cyclase-dependent stablilization of the 3' end of SGLT-1 mRNA (to increase half-life), and SGLT-1 translation and insertion into the apical brush border membrane.

In animals, a wide range of sweet stimuli are capable of upregulating SGLT-1 expression and function, including LCS (45–48), indicating that SGLT-1 activity is modulated by an upstream and broadly tuned sweet taste sensor. Accordingly, STRs may have the capacity to stimulate gut hormone release both directly, and indirectly by augmenting SGLT-1 function. The latter is evidenced in mice lacking T1R3 or α-gustducin, where SGLT-1 expression and function are not increased in response to dietary glucose or LCS supplementation as occurs in control mice (42). Moreover, the 3-fold increase in jejunal SGLT-1 expression following 4 days of sucralose gavage (100 mg, twice-daily) in control mice was absent in our mice lacking both T1R2 and T1R3 subunits of the STR (Marino Z, Young RL; Figure 1). Together, these experiments attest to the importance of intestinal STRs in regulating SGLT-1 function in mice, and support the notion that LCS can potentiate postprandial glycemic excursions via STR-dependent gains in SGLT-1 function and glucose absorption, in response to habitual consumption of sugars or LCS (Figure 2).

There is evidence that enteric neurons link glucose sensing in EEC to glucose transport function in enterocytes (50). Studies in rodents have shown that intestinal areas adjacent to regions exposed to LCS have increased SGLT-1 expression (46). This communication between STR-equipped L cells and SGLT-1-bearing absorptive enterocytes is likely to involve gut hormone intermediaries, such as GLP-1 and/or GLP-2. Indeed, GLP-2 receptors are present on enteric neurons in guinea pig ileum, mouse jejunum, mouse and pig intestine (20, 51, 52) and absorptive enterocyte progenitors in mouse jejunum respond to GLP-2 in an enteric neuron-dependent manner (52). GLP-2 is also capable of upregulating SGLT-1 expression *in vivo* (28), and STR-dependent release of both GLP-1 and GLP-2 is detected at higher concentrations in the portal and lymphatic circulation than the systemic circulation in rodents (28, 53). This indicates that local release of either mediator in response to sweet stimuli, including LCS, may be sufficient to increase SGLT-1 function. It may also, in part, explain the equivocal nature of human data on LCS-evoked gut hormone release, as paracrine signaling in the mucosa could occur in the absence of a substantial contribution to circulating hormone levels. To this end, we provided the first evidence that LCS evoke *ex vivo* GLP-1 release from human ileal tissue (26). However, the precise signal transduction pathways utilized by LCS to trigger gut hormone release in human mucosa remain to be identified.

An increase in SGLT-1 protein in the apical brush border of enterocytes occurs in a cyclic AMP (cAMP)-dependent manner in response to transduction of basolateral signals (54, 55), and secondary to an increase in SGLT-1 transcription (56) and stabilization (increased half-life) of the 3′-untranslated region of the SGLT-1 transcript (57, 58). This facilitates an increase in apical SGLT-1 protein translation and insertion in response to gut hormone signaling. Jugular vein infusion of GLP-2 increases the abundance of SGLT-1 protein and rate of SGLT-1-dependent glucose transport in the apical membrane of jejunal enterocytes in rats, a response abolished when protein translocation is inhibited with brefeldin (29, 59). This highlights the importance of GLP-2 in the regulation of SGLT-1 function at the apical brush border membrane.

While enteric neurons express receptors for other gut hormones, including GIP, GIP is unlikely to be responsible for glucose or LCS effects on SGLT-1 (20, 60). GIP receptor knockout and wild type mice show similar increases in jejunal SGLT-1 expression on a high carbohydrate diet, compared to mice on a low carbohydrate diet (20). Irrespective of which mucosal mediator is a trigger upon intestinal STR activation, the interplay between these broadly-tuned receptors and SGLT-1 is critical for glucose absorption and represents a major mechanism regulating overall glycemic control.

## Type 2 Diabetes is Associated With STR Dysregulation

Globally, over 400 million people are living with diabetes, projected to rise to over 600 million by 2040 (61). Effective control of glycemia, as assessed by glycated hemoglobin (HbA1c) <6.5–7.0% (48–53 mmol/mol), is important to minimize the risk of the development and progression of microvascular complications (i.e., eye, kidney, and nerve damage), and to a lesser extent, macrovascular complications. In the majority of patients with type 2 diabetes, who are relatively well-controlled, postprandial glycaemic excursions predominate over fasting blood glucose levels in contributing to HbA1c (62), and are determined by meal composition, the rate of gastric emptying, hepatic and peripheral glucose metabolism, intestinal glucose absorption, and insulin secretion and resistance (63). Meal-related secretion of insulin is augmented through the insulinotropic actions of the incretin hormones GIP and GLP-1 to reduce postprandial glycemic excursions in health (64, 65); in type 2 diabetes, a markedly attenuated insulinotropic action of GIP (66) and, in some cases, attenuated secretion of GLP-1 (67), contribute to an impairment of postprandial insulin secretion, so that the latter is insufficient to maintain euglycaemia. Furthermore, gut-derived 5-HT can also modulate glucose and energy homeostasis (68–70), and is augmented in patients with type 2 diabetes (71) and the obese (9).

The recognition that the gut, and EEC signals, are major determinants of glycemic control is attested to by the successful deployment of incretin-based therapies for type 2 diabetes. These include mimetics of GLP-1, GLP-1/GIP dual receptor agonists, and inhibitors of dipeptidyl peptidase-IV, which inactivates endogenous GLP-1 (72). These pharmaceutical compounds have improved clinical management of type 2 diabetes substantially, but their use is compromised by cost, compliance with administration, adverse gastrointestinal effects, or suboptimal efficacy in some patients.

While experiments in animal models and patients with type 2 diabetes have shown a gain in function of SGLT-1 and corresponding increase in the rate of intestinal glucose absorption (73, 74), the targeting of intestinal glucose absorption has received comparatively little attention. Indeed, it is likely that a proportion of the clinical benefits of the anti-diabetic gliflozin-class agents (SGLT-2 inhibitors) are due to actions at intestinal SGLT-1. This is particularly true for first-in-class examples, such as the dual SGLT-1/SGLT-2 inhibitor sotagliflozin, which has lower selectivity for SGLT-2 and acts beyond inhibition of renal glucose reabsorption by SGLT-2 to induce partial inhibition of intestinal SGLT-1, leading to augmented GLP-1 and insulin secretion, and a reduction in postprandial glucose excursions (75).

To assess whether regulation of intestinal STR was disrupted in patients with type 2 diabetes, and had an unfavorable impact on glucose absorption and postprandial hyperglycemia, we compared intestinal STR expression in individuals with and without type 2 diabetes. We first established that STRs were expressed at similar levels in the duodenum in both groups when sampled at euglycemia (49). However, we found that T1R2 expression was decreased following enteral glucose exposure under hyperglycemic conditions in non-diabetic subjects, but remained elevated in patients with type 2 diabetes, where it was linked to an increase in glucose absorption (assessed by serum levels of 3-OMG which had been co-administered with the glucose load) (22). These findings support the notion that intestinal STR dysregulation in type 2 diabetes can exacerbate postprandial glycemic excursions. Furthermore, given that patients with type 2 diabetes are 3-fold more likely to consume beverages sweetened with LCS than healthy individuals (76), it is possible that high dietary LCS consumption contributes to, rather than alleviates, postprandial glycemic dysregulation.

## Low-Calorie Sweeteners and Glycemic Control

Sugar-sweetened beverages contain high levels of sucrose or high fructose corn syrup (77) and represent a major source of added sugars in western diets. They account for around 16% of daily caloric intake of adults in the United States (78) and 11% in Canada and Australia (79), a level that exceeds the World Health Organization recommendation that added sugar consumption should be limited to 10% of daily caloric intake (80). These sugars are rapidly absorbed by the small intestine to increase glycemic load, which, when associated with increased peripheral insulin resistance, increases the risk of developing type 2 diabetes (81).

The outcomes of epidemiological studies indicate that high and habitual consumption of sugar-sweetened beverages is associated with an increased risk of developing type 2 diabetes, independent of total energy intake or body mass (77, 82). While these findings do not establish causality (83, 84), the adverse health outcomes linked to high sugar consumption have led to changes in global health policy to limit such intake, with several countries now implementing a sugar tax (80, 85). Not surprisingly, beverages sweetened with LCS have become a popular alternative.

Diet beverages contain a single LCS, or more frequently, LCS combinations, in place of sugars (86), with specific LCS commonly identified by their European Food Safety Authority E-number, i.e., aspartame (E951), sucralose (E955), and acesulfame-K (E950). LCS differ substantially in their oral bioavailability and, therein, exposure to intestinal regions and their microbiota. For example, aspartame is completely hydrolyzed in the proximal intestine to methanol and constituent amino acids, aspartate and phenylalanine, and has no effective oral bioavailability or exposure to the distal intestine and its microbiota. Sucralose has low oral bioavailability (around 15%), but full exposure to the intestine and microbiota due to excretion in largely unchanged form in feces; minor absorbed sucralose and glucoronidation end-products undergo renal excretion. Finally, acesulfame-K has high oral bioavailability (90–100%) due to rapid absorption in the proximal intestine and has limited exposure to the distal intestine and its microbiota; acesulfame-K is cleared via renal excretion in largely unchanged form [for reviews, see (87–89)]. These distinct properties should be considered in interpreting effects of LCS both within, and outside, the gastrointestinal tract.

LCS are 200 to 13,000 times sweeter than sucrose by weight, and were expected to be beneficial in the setting of obesity and type 2 diabetes due to their low calorie content. There is, however, only equivocal evidence of this benefit, with several epidemiological studies indicating little or no benefit, or even an increased risk of weight gain (90–92). Moreover, some epidemiological studies suggest that a high habitual intake of beverages sweetened with LCS is associated with an increased risk of developing type 2 diabetes (93–97). Reverse causality (e.g., people opting for LCS-sweetened beverages in response to weight gain and/or obesity, or subclinical disease including pre-diabetes) is unlikely to fully account for the increased risk, which is evident even after adjusting for differences in body mass and energy intake. Furthermore, two studies have reported an elevated risk of developing type 2 diabetes in normal weight individuals (93, 97).

The outcomes of studies that have prospectively investigated the effects of LCS intake on long-term glycaemic control (assessed by HbA1c) or insulin resistance have been equivocal, and several failed to adjust for differences in sugar intake (76, 98–102). Despite this, high habitual patterns of LCS consumption have been reported to increase HbA1c levels in healthy adults, independent of body mass (101), while daily LCS consumption has been dose-dependently associated with HbA1c increases in type 2 diabetes (76). A negative impact of LCS on acute glycemic control has also been shown in obese individuals, where a sucralose preload consumed in advance of an oral glucose tolerance test augmented blood glucose levels over the following 5 h substantially, when compared to water or no preload (103).

Collectively, the potential for LCS to impair glycemic control remains uncertain, in large part due to the small number of prospective clinical studies (104, 105). Proposed mechanisms linking LCS to an increased risk of developing type 2 diabetes in humans include a reduced fidelity of central responses to nutritive stimuli, effects on gut microbiota, and an effect of LCS to augment glucose absorption.

We recently reported early findings of a randomized placebo-controlled clinical study investigating the effect of diet supplementation with combined LCS (sucralose 276 mg, acesulfame-K 156 mg in capsules; equivalent to 1.5 L diet beverage/day) over 2 weeks on glycemic responses to enteral glucose. We observed a clinically significant effect of LCS to increase the rate of glucose absorption and augment blood glucose responses to enteral glucose in healthy subjects consuming LCS, relative to placebo. Moreover, glucose-evoked GLP-1 and GLP-2 release was decreased in LCS-consuming participants, which may relate to the more rapid proximal absorption of glucose limiting the exposure of more distally located L-cells (106). These findings indicate a negative impact of habitual high LCS intake on glucose absorption and acute glycaemic control in health, and add support for the concept that high habitual intake of LCS may increase the magnitude of postprandial glycemic excursions.

## LCS and the Gut Microbiome

The gut microbiome comprises the diverse range of bacteria, yeasts, and other microorganisms which exist in a largely symbiotic relationship with the host (107). These prevent potentially harmful microorganisms from colonizing the gut by competing for energy resources (108). Use of these resources liberates nutrients which would be otherwise inaccessible to the host, i.e., microbial conversion of indigestible polysaccharides to short chain fatty acids (SCFAs) such as acetate, propionate, and butyrate, which act as substrates for cellular metabolism, gluconeogenesis and lipogenesis. Moreover, SCFAs play a crucial role in satiety signaling, and modulate appetite directly and indirectly via leptin synthesis in adipose tissue (109). SCFAs also have a beneficial impact on glycemia, with propionate shown to improve insulin sensitivity, and butyrate to prevent or improve insulin resistance in mice fed a high fat diet (110–112). Microbial-derived signals from the gut, therefore, have the potential to influence glycemic control substantially.

The composition of the gut microbiome of individuals with type 2 diabetes differs from that of non-diabetic individuals, in its relative and BMI-independent decrease in abundance of species from the *Clostridium* phylum (113, 114). These species are negatively correlated with markers of poor glycemic control such as fasting glucose, HbA1c and insulin, but positively correlated with the insulin sensitizing hormone adiponectin (113). Alterations in the gut microbiome of individuals with type 2 diabetes are also associated with changes in functional microbial genes, with a specific enrichment of pathways for starch, glucose, fructose, and mannose metabolism, which increases the potential for energy harvest and metabolism (113). These changes are causally related to the development of insulin insensitivity and resistance, as allogenic transplantation of intestinal microbiota from lean donors to recipients with the metabolic syndrome improved insulin sensitivity (115). This highlights the importance of the gut microbiome composition with respect to the development of metabolic disorders, including type 2 diabetes.

Exposure to LCS has been shown to drive glucose intolerance in mice via a LCS-dependent shift in composition of the gut microbiome (“dysbiosis”). Transplantation of fecal microbiota from donor mice supplemented chronically with LCS (saccharin) to germ-free recipient mice resulted in glucose intolerance after 6 days. Changes in abundance of more than 40 operational taxonomic units were demonstrated in the recipient mice, along with an upregulation of microbial carbohydrate-related metabolic pathways, and an increase in fecal SCFA levels (101). This increase in SCFAs was speculated to represent increased microbial energy harvest, but may equally represent the outcome of differences in intestinal transit time or absorption (116, 117). It is also been unclear whether fecal bacterial samples accurately represent the microbiome of the proximal gut (118). Indeed, Daly et al. showed that supplementation with SUCRAM (neohesperidin dihydrochalcone and saccharin) over 2 weeks in weaned piglets increased the abundance of *Lactobacillaceae* in cecal, but not fecal, samples, while cecal SCFA levels were comparable in the LCS and control diet groups (117). These findings underscore the importance of testing regional (or mucosa-associated) bacteria in the gut, and of establishing causal mechanisms as opposed to microbial followers of changes in host metabolism.

Causal mechanisms linking dysbiosis to impaired GLP-1 signaling in the gut-brain axis were recently investigated in mouse models of diet-induced type 2 diabetes. Grasset et al., identified a subset of ileal bacteria in these mice that disrupted GLP-1-dependent nitric oxide production in ileal enteric neurons via an attenuation of GLP-1 receptor expression, and showed that this drove GLP-1 resistance in the regulation of gastric emptying and insulin release (119). A GLP-1 resistant phenotype in germ-free mice was rescued through conventionalization with ileal bacteria from control-fed mice, but not from mice fed the diabetogenic diet, while antibiotic treatment led to GLP-1 resistance in control-fed mice, but improved GLP-1 resistance in diabetogenic diet-fed mice. This study demonstrated that diabetogenic diet-induced gut dysbiosis was causally related to dysglycemia via disruption of GLP-1 signaling in the gut-brain axis, but did not extend to an assessment of specific bacterial populations or products that mediated this effect.

Accordingly, clinical studies are now required to determine whether LCS induce intestinal dysbiosis in humans, whether this is causally related to disruption of the gut-brain axis that controls glycemia, and which microbiome-derived signals effect this change. Such investigation holds the potential to usher in new classes of anti-diabetic therapy which would correct defects in microbiome composition and/or associated signaling pathways that impact glycemic control adversely.

## Tasting Sweet Via Non-STR Pathways

Several studies have reported the existence of a lingual and STR-independent sensor tuned to detect the nutritive value of complex carbohydrates. Behavioral studies in rodents have shown that rats prefer consumption of polycose (glucose oligomer) solutions above that of water or solutions of the disaccharides sucrose and maltose, particularly at low concentrations (120, 121). This was further supported by electrophysiology studies of lingual nerve activity, which indicated that rats could distinguish the tastes of polycose and sucrose (122, 123). Importantly, mice lacking one or both STR subunits had limited, or no behavioral or lingual nerve responses to simple sugars, while responses to polycose remained normal (124–127). More recently, behavioral research on human taste detection have added support for a human polycose taste receptor, showing that humans can detect glucose oligomer solutions on an equimolar basis to simple sugars, even when lingual STR were blocked with lactisole and amylase activity inhibited by an α-glucosidase inhibitor (to prevent oral breakdown of glucose oligomers to STR-detectable mono- and disaccharides) (128–130). The latter study also indicated that oligosaccharides of 4 or higher degrees of polymerization (i.e., maltotetraose) were detected by a STR-independent lingual taste pathway in humans. While a polycose receptor is yet to be cloned, future characterization may also reveal its potential as an intestinal nutrient sensor, and whether there are associated consequences for glycemic control in humans.

## Conclusion

**Figure 3:**
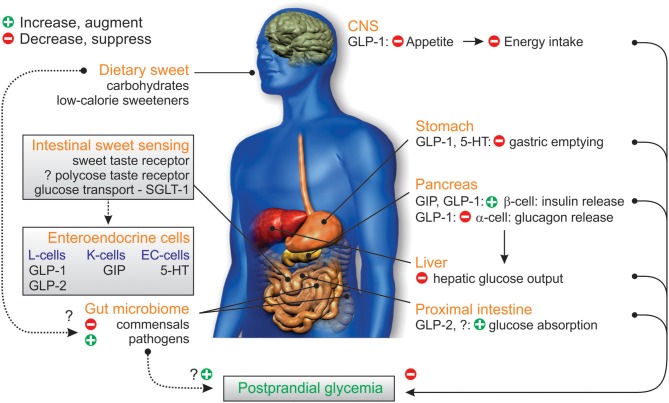
Gastrointestinal factors influencing glycemic control. Dietary sweet stimuli can activate STR in the proximal intestine facilitating the enteroendocrine cell release of the incretin peptides GIP from K-cells and GLP-1 from L-cells, as well as 5-HT from EC-cells; substrates of SGLT-1 (glucose, galactose) also trigger GIP and GLP-2 release. GIP and GLP-1 stimulate glucose-dependent insulin release, to increase glucose disposal; GLP-1 and 5-HT also slow the rate of gastric emptying via vagus nerve signals (not shown) while GLP-1 inhibits pancreatic glucagon release, leading to reduced hepatic glucose output. GLP-2 co-released from L-cells acts to increase intestinal glucose absorption via an increase in the capacity for SGLT-1-based glucose transport. Dietary sweet stimuli can also alter the composition of the gut microbiome in favor of colonization of gut pathogens over fermentative gut commensals, which can affect energy harvest, and disrupt microbiome signaling to the host and glycemic control. Together these influences can disrupt the homeostatic balance between glucose-evoked gut hormone release, glucose absorption, and microbiome composition, leading to dysglycemia which would potentially be harmful in the setting of type 2 diabetes. In addition, complex carbohydrates (oligosaccharides) may contribute to these processes via a yet to be identified polycose taste receptor.

Although foods and beverages sweetened with LCS have become a popular alternative to their sugar-sweetened counterparts, research relating to their impact on acute and chronic human health has been inappropriately limited, and the outcomes equivocal. However, the outcomes of the hitherto small number of well-conducted studies raises concerns regarding their health impact. Further research is now required to better characterize the EEC biology of intestinal sweet taste signaling in humans, characterize the mechanisms utilized by LCS to impact glycemic control, and identify potential targets capable of modifying STR signaling for clinical benefits (Figure 3). In addition, studies are needed to determine whether patterns of LCS consumption can trigger gut dysbiosis, with consequences for human health as are subsequent metagenomic, metabolomic, and functional investigations of causal mechanisms. These hold the high potential for improved prevention and novel management of type 2 diabetes.

## Author Contributions

DK, DJK, TW, MH, CR, and RY were all involved in conception, design, and writing of the manuscript. All authors have approved the publication of this final version of the manuscript.

### Conflict of Interest Statement

The authors declare that the research was conducted in the absence of any commercial or financial relationships that could be construed as a potential conflict of interest.
